# Sustained antigen delivery improves germinal center reaction and increases antibody responses in neonatal mice

**DOI:** 10.1038/s41541-024-00875-3

**Published:** 2024-05-25

**Authors:** Leda Lotspeich-Cole, Swetha Parvathaneni, Jiro Sakai, Lunhua Liu, Kazuyo Takeda, Robert C. Lee, Mustafa Akkoyunlu

**Affiliations:** 1 US FDA/CBER/OVRR/DBPAP, 10903 New Hampshire Avenue, Silver Spring, MD USA; 2 US FDA/CBER/OBRR/DBCD, 10903 New Hampshire Avenue, Silver Spring, MD USA

**Keywords:** Conjugate vaccines, Conjugate vaccines

## Abstract

Neonates and young infants are known to have limited responses to pediatric vaccines due to reduced germinal center formation. Extended vaccine antigen dosing was previously shown to expand germinal center formation and improve humoral responses in adult mice. We report that sustained antigen delivery through sequential dosing overcomes neonatal limitations to form germinal center reactions and improves humoral immunity. Thus, vaccine strategies that extend the release of vaccine antigens may reduce the number of doses, and time needed, to achieve protective immunity in neonates and young infants.

## Introduction

Most pediatric vaccines are administered three or more times during the first 15 months of life to achieve protective immunity^1^. Until the immunizations are complete, infants are at increased risk of infection. In a study on completion and compliance of childhood vaccinations in the United States, only 26% of children received all doses of six recommended vaccines on time^2^. Of the 74% of children who received at least one late dose, 39% were under-vaccinated for over 7 months. Simplifying vaccine schedules would allow protection earlier and reduce under-vaccination.

Most vaccines, including the pneumococcal conjugate vaccines, generate long-term immunity by eliciting antibody (Ab)-secreting long-lived plasma cells (LLPCs) and memory B cells (MBCs)^3,4^. High-affinity vaccine antigen-specific LLPCs and MBCs are formed during germinal center (GC) reactions, where GC B cells undergo affinity maturation and then differentiate into MBCs or LLPCs. The GC reaction is regulated by specialized T follicular helper (T_FH_) cells, which regulate the proliferation, survival, and differentiation of GC B cells through the delivery of cytokines and costimulatory molecules^5–8^. As germinal centers are central to the development of high-affinity plasma cells and memory B cells, they are a well-studied aspect of vaccination studies. Neonates and young infants are known to have limited GC formation after vaccination, resulting in few T_FH_ cells, MBCs, and LLPCs, as well as reduced isotype-switched antibody levels^9–11^.

GC lifespan is also dependent on the nature of the immune stimulus. For example, model protein antigens adjuvanted with alum induce short-lived GCs that resolve within a month while viral infections can lead to GC B cell proliferation for at least 3 months^12–14^. Long-lived GCs allow affinity maturation of B cells and the development of durable, specific, humoral responses. Because of the timeline of GC development and antibody optimization, immunologists have postulated that antigen kinetics could have a profound effect on the magnitude and quality of germinal center responses and long-term immunity^15^. During natural infection, the immune system is exposed to escalating antigen doses for days to weeks, whereas most non-live vaccines are single bolus events. Mimicking the natural infection, administering a given total dose of antigen and adjuvant over 1–2 weeks through repeated injections or osmotic pump enhanced GC formation and humoral responses in adult mice^15,16^. Here, we explored whether sustained antigen delivery improves neonatal mice’s humoral immune responses to a model pneumococcal polysaccharide conjugate vaccine consisting of pneumococcal polysaccharide type 14 conjugated to tetanus toxoid protein (PPS14-TT) with an alum adjuvant.

## Results

### Sustained delivery of PPS14-TT improves antibody responses against the vaccine

**Fig. 1:**
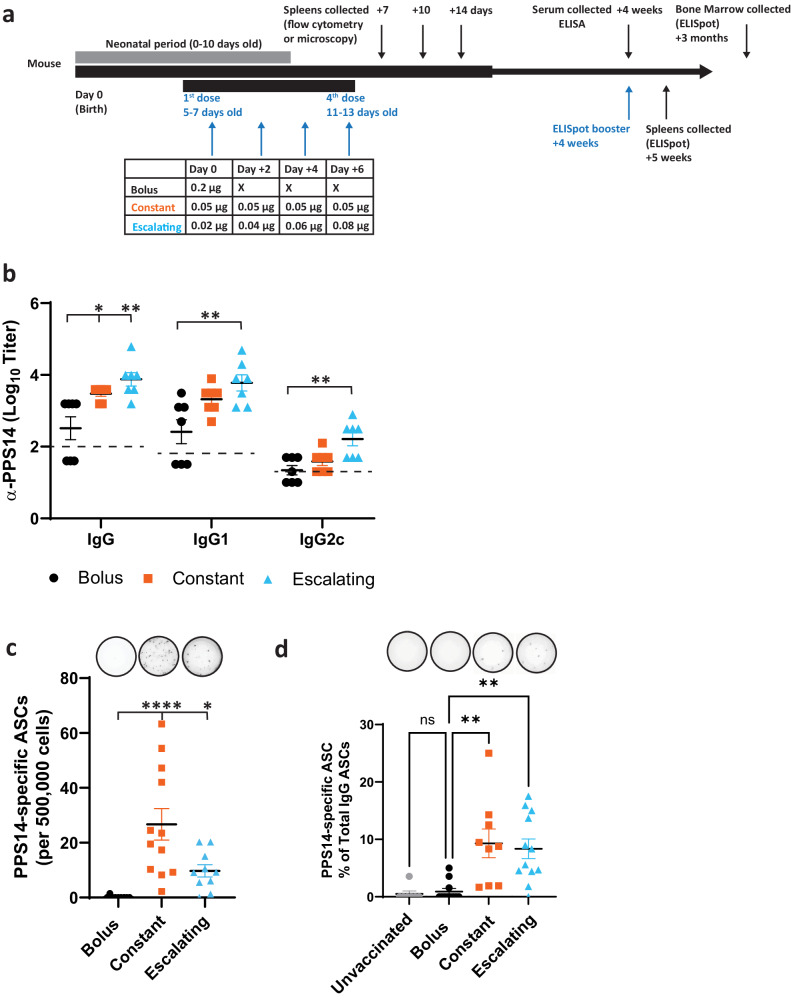
Extending antigen availability through sequential dosing increases antibody responses after vaccination in neonatal mice. **a** Groups of neonatal C57BL/6 mice were immunized with a total of 0.2 μg of PPS14-TT + 25% by volume Imject^TM^ following the dosing schedules shown and the immune responses were evaluated on the indicated time points. **b** Serum total IgG as well as IgG1 and IgG2c antibodies against PPS14 were measured by ELISA following s.c. immunization of mice (n = 7 mice per group). **c** Mice were immunized s.c. with PPS14-TT and boosted 4-weeks later. Seven days after boosting, splenocytes were collected to measure PPS14-specific IgG+ ASCs by ELISPOT. (bolus *n* = 9; constant *n* = 12; escalating *n* = 10). **d** Bone marrow was harvested 3-months after i.p. immunization with PPS14-TT to measure PPS14-specific IgG+ ASCs by ELISPOT (bolus *n* = 11; constant *n* = 9; escalating *n* = 12; unimmunized *n* = 7). Data are representative of experiments done twice (**b**); or in **c** and **d**, data were pooled from two independent experiments. Mean $\pm \,$SEM is shown, and each data point represents an individual mouse. Asterisk indicates statistically different from bolus as determined by Kruskal–Wallis one-way ANOVA with Dunn’s multiple comparisons test. *p $\le$ 0.05, **p$\,\le$ 0.01, ****p$\,\le$ 0.0001.

To investigate the effects of sustained vaccine delivery in neonatal mice, we measured anti-PPS14 IgG titers 4-weeks after vaccinating 5–7-day-old C57Bl/6 mice with a single bolus dose, four constant doses, or four escalating doses of a PPS14-TT conjugate vaccine (Fig. 1a, b). We detected a significantly higher PPS14-specific total IgG in mice receiving the constant or escalating dose series than the bolus group. PPS14-specific IgG1 and IgG2c titers were also elevated in mice receiving the constant or escalating doses compared to those that received a bolus dose but only the escalating group response was statistically significantly higher than the bolus group. Of note, approximately half of the mice receiving a bolus dose had no measurable response to the PPS14-TT vaccine while all mice immunized with the constant or escalating dose series had a measurable response to the vaccine. Higher antibody titers observed in the sustained delivery groups could not be explained by the fact that the mice were older when they received their final doses of vaccine because 13-day-old mice receiving vaccinations corresponding to the final doses of the constant or escalating series, or the 0.2 µg bolus dose, had responses that did not substantially differ from 5-day-old mice receiving the bolus dose (Supplementary Fig. 1).

### Sustained delivery of PPS14-TT improves antibody-secreting cell (ASC) responses against the vaccine

In support of sustained antigen delivery improving neonatal antibody titers, mice receiving the constant and escalating doses of the vaccine had substantially more PPS14-specific IgG antibody ASCs than mice receiving the bolus dose (Fig. 1c, d). In the spleen, only one mouse that received a bolus dose had detectable PPS14-specific ASCs 4-weeks after vaccination, while all mice receiving the constant dose had detectable PPS14-specific ASCs after vaccination (Fig. 1c). Interestingly, mice receiving the escalating dose series had fewer detectable PPS14-specific ASCs than mice receiving the constant series but only one mouse had no detectable PPS14-specific ASCs. Additionally, we compared long-lived plasma cell numbers in the bone marrow 3-months after vaccinating 5–7-day-old C57Bl/6 mice with a single bolus dose, four constant doses, or four escalating doses of a PPS14-TT conjugate vaccine (Fig. 1a, d). PPS14-specific ASCs were significantly higher in mice that had received the constant dosing or escalating dosing regimens compared to the bolus-dose mice. Neonates receiving bolus doses had very few PPS-14-specific ASCs in the bone marrow and ASC numbers were not significantly higher than in the unvaccinated controls. These experiments provide evidence that sustained antigen delivery can overcome weak humoral responses to vaccination in neonates as was shown previously in adult mice^15^.

### Sustained delivery of PPS14-TT improves splenic GC response

**Fig. 2:**
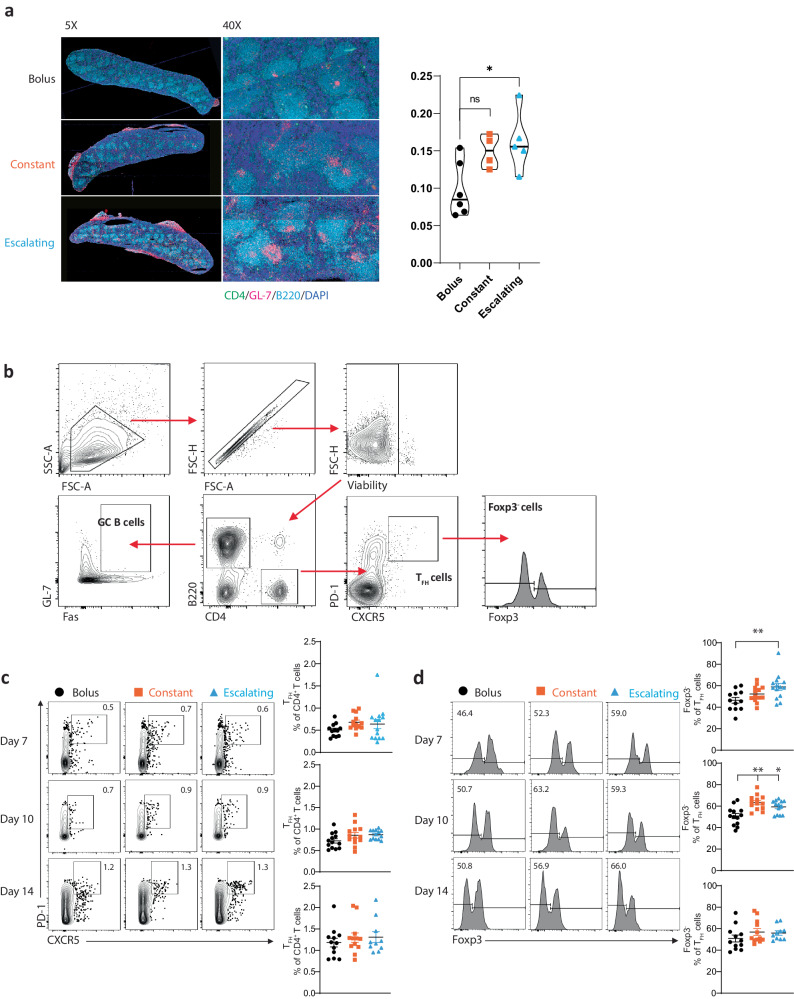
Sequential dosing increases GC responses after vaccination in neonatal mice. Mice were i.p. immunized with PPS14-TT. Splenic GC responses were measured by microscopy at 10-days post-vaccination and by flow cytometry 7, 10, and 14-days post-vaccination. **a** Representative immunofluorescence staining images of spleens from neonatal mice immunized according to bolus, constant, and escalating schedule. Sections were stained with CD4, GL-7, and B220 antibodies in addition to Hoechst counterstaining. Violin plots show mean $\pm \,$SEM GC center counts. *p $\le$ 0.05. **b** Representative gating strategy for splenic T_FH_, Foxp3^-^ T_FH_, and GC B cells. **c** Representative contour plots of T_FH_ cells with mean percent of CD4^+^ T cells (right), and frequency of T_FH_ cells as a percent of CD4^+^ T cells (left). Cells were pregated on live dump^-^CD4^+^ B220^-^ T cells. **d** Representative histograms of Foxp3^-^ T_FH_ with mean percent of T_FH_ cells (right), and frequency of Foxp3^-^ cells (left). Cells were pregated on live dump^-^CD4^+^ B220^-^PD-1^+^ CXCR5^+^ T cells. In **c** and **d**, on Day 7: bolus dose *n* = 12 mice; constant dose *n* = 13 mice; escalating dose *n* = 14 mice. On Day 10: bolus dose *n* = 13 mice; constant dose *n* = 13 mice; escalating dose *n* = 13 mice. On Day 14: bolus dose *n* = 11 mice; constant dose *n* = 12 mice; escalating dose *n* = 11 mice. Data were combined from two independent experiments (**c**–**e**). Mean $\pm \,$SEM is shown, and each data point represents an individual mouse. Kruskal–Wallis one-way ANOVA with Dunn’s multiple comparisons test was used for statistical evaluations. *p $\le$ 0.05, **p$\,\le$ 0.01.

To determine whether sustained antigen delivery could boost GC formation in neonatal mice as previously described in adult mice^15^, neonatal mice were immunized intraperitoneally (i.p.) as described in Fig. 1a and splenic GC formation was evaluated 10 days after the bolus dose using antibodies against CD4, GL-7 and B220. Properly formed GCs were mostly visible in sequentially administered neonatal mice (Fig. 2a). In bolus-immunized mice GCs were minimally observed. Quantification of the number of GC numbers revealed that the escalating dose group had significantly higher numbers of GCs than the bolus dose group; however, the increase GC number in the constant dose group did not reach statistical significance when compared to bolus-immunized mice. Splenic T_FH_ cell responses were examined by flow cytometry 7, 10, and 14 days after initiating vaccination (Fig. 2b, c, d). When we gated CD4^+^PD-1^+^CXCR5^+^ cells (Fig. 2b), we found no significant difference between the immunization groups (Fig. 2c). However, further gating out Foxp3^+^ follicular regulatory cells indicated that the proportion of Foxp3^-^ T_FH_ cells were elevated in neonates receiving sequential doses of the PPS14-TT vaccine compared to the bolus dose (Fig. 2d). Foxp3^-^ T_FH_ proportions peaked on day 10, with significantly elevated Foxp3^-^ T_FH_ proportions observed in the constant and escalating groups.

T_FH_ cells provide help to B cells in GCs, and we observed a larger number of antigen-specific ASCs in neonates immunized with constant and escalating doses of the PPS14-TT vaccine (Fig. 1c, d), so we hypothesized that the sequential doses would also induce stronger GC B cell responses in the spleen. The constant and escalating dose schedules enhanced the differentiation of GC B cells (Fas^+^GL7^+^) (Fig. 2b and Supplementary Fig. 2). A significant increase in GC B cells was observed at day 10 for the group receiving a constant dose series (Supplementary Fig. 2). By day 14, GC B cell numbers had decreased, and there was no difference between the three groups. These data demonstrate that four doses of the PPS14-TT vaccine over the course of 6 days induce superior T_FH_ and GC B cell responses compared to a single bolus dose.

## Discussion

This study demonstrates that neonatal humoral immunity and GC development after vaccination are improved by sustained antigen delivery through sequential administration of the PPS14-TT vaccine.

Although sequential administration of vaccines is of limited practical use in humans, emerging vaccine technologies could be applied to early childhood vaccines. Sustained antigen expression may partially explain the success of the SARS-CoV-2 mRNA vaccines since they can lead to antigen expression for approximately 10 days in mice^17^, which is longer than the 6-day vaccination period used in this study. Like studies using sustained antigen delivery in mice, humans immunized with two doses of the SARS-CoV-2 mRNA-based BNT162b2 vaccine are shown to elicit high frequencies of antigen-binding GC B cells in draining lymph nodes for at least 12 weeks after boosting^18^. This suggests that like sustained antigen delivery via osmotic pump^16,19^ or sequential administration of vaccine^15^, SARS-CoV-2 mRNA-based vaccines can induce persistent GC B cell responses enabling the generation of robust humoral immunity^18^. In addition to mRNA vaccines, the use of short phosphoserine peptide linkers that promote stable binding of immunogens to alum has also been shown to enhance humoral immunity through extended antigen availability in lymph nodes in adult mice^20,21^. Thus, improved humoral immune response accompanied by enhanced GC following sequential administration of the vaccine in neonatal mice serves as a proof of concept and suggests that antigen delivery systems facilitating sustained antigen availability in lymph nodes might also improve vaccine responses at an early age.

## Methods

### Mice

C57BL/6 genetic background breeding pairs were purchased from Jackson Laboratory. All animal procedures were approved by the FDA Institutional Animal Care and Use Committee (Protocol 2002-37 and Protocol 2017-48). For neonatal mouse immunization experiments, 5–7-day-old mice were used. For euthanasia, adult and neonatal mice were exposed to CO_2_ inhalation. CO_2_ was introduced at a rate of at least 30% chamber volume per minute. CO_2_ inhalation was followed by cervical dislocation for adult mice and decapitation for neonatal mice. Anesthesia was induced by administering isoflurane, set at 3–4% for 1–2 min in the induction box and the flow rate of O_2_ is set at 1.0 L/min.

### Immunization

Neonatal (5–7-day-old) mice were immunized subcutaneous (s.c.) or i.p. PPS14-TT vaccine was manufactured as described previously^22^. The PPS14 to TT ratio in the PPS14-TT vaccine is 1:14 (w/w). PPS14-TT vaccine was emulsified with 25% by volume Imject^TM^ Alum (Thermo Scientific, Rockville, MD). Injection volumes were 30 μL.

### Enzyme-linked immunosorbent assay (ELISA)

For antibody measurement, 96-well plates were coated with PPS14 (197-X) (ATCC, Manassas, VA) at 10 μg/mL in PBS (pH 7.4) for 2 h at room temperature. After washing, plates were blocked with 5% NBCS (Gibco, Gaithersburg, MD) in PBS (pH 7.4) for 30 min. Blood was collected by terminal cardiac puncture or by tail-bleed, and serum was processed by spinning 10 min at 5000 x *g* at 4 ^o^C. Serum was used immediately or stored at –80^ o^C. For total IgG, serum was diluted 1:100 in 5% NBCS in PBS; for IgG1, serum was diluted 1:80; and for IgG2c, serum was diluted 1:20. Next, serum was serially diluted and 100 μL of diluted samples were transferred on coated plates for overnight incubation at 4 ^o^C. After washing, wells were incubated for 3 h at room temperature with the appropriate horseradish peroxidase-conjugated goat anti-mouse conjugate: for total IgG, goat anti-mouse IgG-Fc (Bethyl, 1:10,000, Cat# A90-131P); for IgG1, goat anti-mouse IgG1 (Bethyl, 1:10,000, Cat# A90-105P); and for IgG2c, goat anti-mouse IgG2c (Bethyl, 1:5,000, Cat# A90-136P). KPL SureBlue TMB Microwell Peroxidase Substrate (1-component) (SeraCare Life Sciences, Gaithersburg, MD) was added, and the optical density (OD) values were obtained with a Synergy HT microplate (BioTek, Vinooski, VT). Endpoint titers were calculated for each sample. Endpoints were calculated using a positive control run on each plate for normalization. In short, the positive OD threshold was calculated as the OD before the positive control curve became linear that was at least twice the OD of the negative control wells. The endpoint titer for each sample was calculated to be the last dilution with an OD above the positive control threshold OD. To normalize between plates, the same positive control dilution was used to find the threshold OD for each plate.

### Splenic ELISPOT

PPS14-specific ASCs in the spleens of PPS14-TT vaccinated neonatal mice were identified by ELISPOT. Neonatal mice were vaccinated with PPS14-TT vaccine and then given a single booster dose of 1 μg PPS14-TT vaccine with 25% by volume Imject^TM^ Alum (Thermo Scientific) in 500 μL, 7-days before spleens were harvested. Briefly, 96-well plates (Millipore, Rockville, MD) were treated with 35% ethanol for less than 1 min and then washed with PBS. Plates were coated with 20 μg/mL PPS14 (197-X) (ATCC) or Goat anti-mouse IgG-Fc Fragment (Bethyl, 1:100, Cat# A90-131A) overnight at 4°C. After washing with PBS, the wells were blocked with B cell medium (RPMI 1640 GlutaMAX Gibco, 10% FBS H/I, 5 mL HEPES, 5 mL MEM, 5 mL sodium pyruvate, 5 mL P/S antibiotic, 500 μL β-mercaptoethanol). Single-cell suspensions from spleens were prepared in RPMI 1640 GlutaMAX (Gibco) with 1% NBCS (Gibco). Serial dilutions of 3 million cells, 2 million cells, and 1 million cells were plated and cultured for 5 h. After washing, wells were incubated with Goat anti-mouse IgG-Fc HRP (Bethyl, 1:5,000, Cat# A90-131P) in 5% NBCS (Gibco) in PBS (pH 7.4) overnight at 4°C. AEC substrate (3-amino-9-ethylcarbazole) (Vector laboratories) was added after thoroughly washing and plates were incubated at room temperature in the dark for 10 min. Spots were detected by removing the plate backing, washing 20 times with DI water, and drying the plates in the dark. Spots were identified and counted using a CTL S6 Core Analyzer running ImmunoSpot 7.0.24.1 (ImmunoSpot, Cleveland, OH).

### Bone marrow ELISPOT

PPS14-specific ASCs in the bone marrow of PPS14-TT vaccinated neonatal mice were identified by ELISPOT. Neonatal mice were vaccinated with the PPS14-TT vaccine. Briefly, 96-well plates (Millipore, Rockville, MD) were treated with 35% ethanol for less than 1 min and then washed with PBS. Plates were coated with 20 μg/mL PPS14 (197-X) (ATCC) or 1:100 Goat anti-mouse IgG-Fc Fragment (Bethyl, 1:100, Cat# A90-131A) overnight at 4 °C. After washing with PBS, the wells were blocked with RPMI 1640 GlutaMAX (Gibco) with 1% NBCS (Gibco). Single-cell suspensions from bone marrow were prepared in RPMI 1640 GlutaMAX (Gibco) with 1% NBCS (Gibco). For PPS14-coated wells, cells were added at 500,000 cells per well or 125,000 cells were added to wells coated with Goat anti-mouse IgG-Fc Fragment. Plated cells were cultured for 5 h. After washing, wells were incubated with 1:5,000 Goat anti-mouse IgG-Fc HRP (Bethyl, 1:5,000, Cat# A90-131P) in 5% NBCS (Gibco) in PBS (pH 7.4) overnight at 4°C. To develop the plates, plates were washed with PBS-T, and AEC substrate (3-amino-9-ethylcarbazole) (Vector laboratories) was added. Plates were incubated at room temperature in the dark for 10 min. Spots were detected by removing the plate backing, washing 20 times with DI water, and drying the plates in the dark. Spots were identified and counted using a CTL S6 Core Analyzer running ImmunoSpot 7.0.24.1 (ImmunoSpot, Cleveland, OH).

### Immunofluorescence microscopy

OCT-embedded spleens were snap-frozen by floating on dry ice-cooled isopentane. Frozen blocks were sectioned at 10 μm and mounted on charged slides and stored at –80 °C. Sections were allowed to air dry at room temperature for 10 min and fixed with pre-cooled Methanol for 10 min, followed by washing three times with PBS. Sections were first blocked with 10% normal donkey serum for 30 min and then incubated with primary antibodies overnight at 4 °C with anti-B220 (ThermoFisher, Rat IgG, 1:50, Cat# 14-0460-82), anti-GL7 (BioLegend, Rat IgM, 1:50, Cat# 144601), CD4-biotin (BD Pharmingen, 1:50, Cat# 553728) antibodies. Following three washing steps with PBS, sections were incubated for 2 h at room temperature with the following secondary antibodies: donkey anti-rat IgG-AF647 (Jackson ImmunoResearch, 1:200, Cat# 712-605-153), anti-rat IgM-AF594 (BioLegend, 1:200, 408912), and streptavidin-AF488 (ThermoFisher, 1:100, Cat# S11223) respectively. Counterstaining was performed by Hoechst 33258 (ThermoFisher, 1:5000, Cat# H3569).

The stained slides were scanned by Nanozoomer XR slide scanner (Hamamastu Photonics, Shizuoka, Japan) for 4-channel fluorescence imaging with filter sets of Hoechst (359/461) [ex/em]; Alexa 488 (499/519); Alexa594(570/590); Alexa 647(652/668). The acquired images were saved in ndpi format for further analysis. Image analysis was performed by NDP.view 2 plus software (Hamamastu Photonics) for counting the number of B220^+^ B cell follicles and GL-7^+^B220^+^ GC formed B cell follicles in the entire spleen. Statistical analysis was performed by Prism 9 (GraphPad, Boston MA) for Mann–Whitney *t*-test and created a violin plot.

### Flow cytometry

Single-cell suspensions were prepared from spleens and stained with fluorochrome-conjugated monoclonal antibodies. To stain dead cells, the suspensions were incubated with eBioscience Fixable Viability Dye eFluor 455UV (ThermoFisher Scientific, 1:1000, Cat# 65-0868-14) or Zombia Aqua Fixable Viability Kit (BioLegend, 1:2000, Cat# 77143) diluted in PBS for 10 min at room temperature. Cells were washed and stained using FACS buffer containing 2% FBS, and 0.5 mM EDTA in PBS. The following antibodies were used for surface staining at room temperature for 20 min, anti-mouse CD4-PerCP-Cy5.5 (BioLegend, 1:100: clone GK1.5, Cat# 100434), anti-mouse B220-BV605 (BioLegend, 1:100, clone RA3-6B2, Cat# 103244), anti-mouse PD-1-PE (BioLegend, 1:100, clone 29 F.1A12, Cat# 135206), anti-mouse CXCR5-biotin (BD Pharmingen, 1:100, clone 2G8, Cat# 551960), anti-mouse CD95-PE-Cy7 (BD Pharmingen, 1:100, clone Jo2, Cat# 557653), and anti-mouse GL7-Alexa488 (BioLegend, 1:100, clone GL7, Cat# 144612). To detect biotinylated CXCR5, cells were further incubated with streptavidin-BV421 (BD Biosciences, 1:500, Cat# 563259) for 15 min at room temperature. For intracellular staining of Foxp3, samples were fixed with Foxp3 Fix/Perm buffer set following the manufacturer’s instructions (eBioscience, Cat# 00-5523-00). Samples were then stained with anti-mouse Foxp3-Alexa Fluor 647 (BD Biosciences, 1:100, clone MF23, Cat# 560401) overnight at 4°C. Flow cytometry data were acquired on an LSR Fortessa flow cytometer (BD Biosciences) and analyzed using FlowJo software v10.7 (TreeStar Inc, Woodburn, OR).

### Statistical analysis

Data were plotted and analyzed with GraphPad Prism v8 (GraphPad Software, Boston, MA). Comparison of antibody responses to the bolus control was calculated using the Kruskal–Wallis one-way ANOVA with Dunn’s multiple comparisons test. ASC levels in the sequential dose groups were compared to the bolus control using the Kruskal–Wallis one-way ANOVA with Dunn’s multiple comparisons test. The effects of sequential dosing compared to the bolus control on T_FH_, T_FR_, and GC B cell levels were calculated using a Kruskal–Wallis one-way ANOVA with Dunn’s multiple comparisons test. *p*-value < 0.05 was considered statistically significant.

### Reporting summary

Further information on research design is available in the Nature Research Reporting Summary linked to this article.

### Supplementary information

Supplemental results and methods

Reporting Summary

## Data Availability

All data generated or analyzed during this study are included in this published article (and its supplementary information files).
