# Supplementary material for: Sustained antigen delivery improves germinal center reaction and increases antibody responses in neonatal mice
Source: NPJ Vaccines. 2024 May 25;9:92. doi: 10.1038/s41541-024-00875-3 (PMC11128021; doi:10.1038/s41541-024-00875-3)
Supplement: Supplementary file 1 — Supplemental results and methods [file 41541_2024_875_MOESM1_ESM.docx]

**Supplemental Results**

**
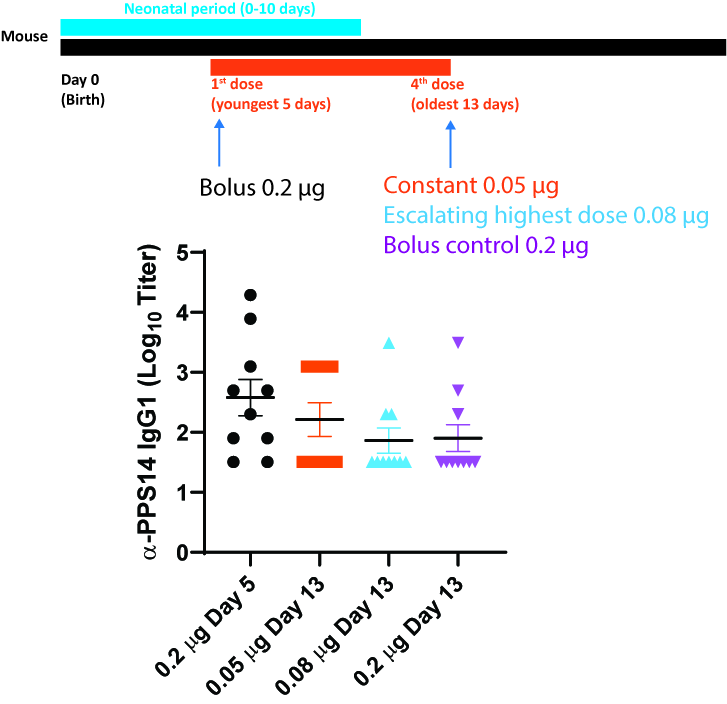
Supplemental Fig. 1. Administering a bolus dose during the neonatal window does not explain weaker responses to bolus vaccination.**

Neonatal C57BL/6 mice were s.c. immunized at 5 or 13 days of age with PPS14-TT + 25% by volume Imject. Five-day-old or 13-day-old mice received a standard bolus dose of 0.2 µg PPS14-TT. Thirteen-day-old mice received a 0.05 µg dose or a 0.08 µg dose corresponding to the last dose of the constant or escalating series respectively. Serum was collected four-weeks later, and anti-PPS14 IgG1 titers were analyzed by ELISA. Data shown are pooled from two independent experiments. For 0.2 µg Day 5 n = 10, 0.05 µg Day 13 n = 9, 0.08 µg Day 13 n = 10, and 0.2 µg Day 13 n = 10. Statistical difference from 0.2 µg Day 5 was determined by Kruskal-Wallis one-way ANOVA with Dunn’s multiple comparisons test.

**Supplemental Fig. 2. Splenic germinal center B cell responses after constant, escalating and bolus immunization of neonatal mice.**

**
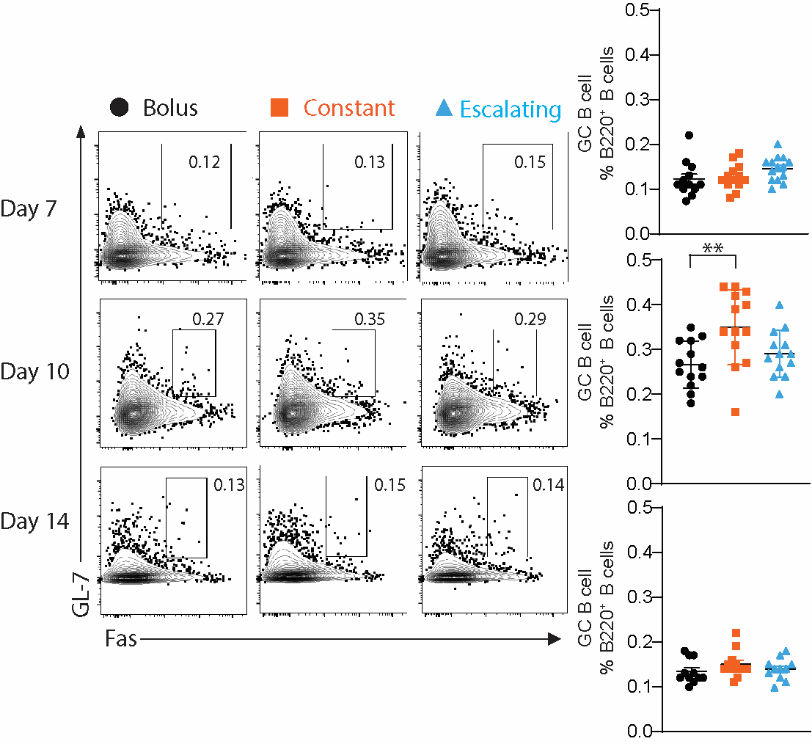
**

Mice were immunized with PPS14-TT i.p.. Splenic GC responses were measured by flow cytometry 7, 10, and 14-days post-vaccination. Representative contour plots of GC B cells with mean percent of B cells in gated population, and frequency of GC B cells (right). Cells were pre-gated on live dump^-^CD4^-^ B220^+^ B cells. On Day 7: bolus dose n = 12 mice; constant dose n = 13 mice; escalating dose n = 14 mice. On Day 10: bolus dose n = 13 mice; constant dose n = 13 mice; escalating dose n = 13 mice. On Day 14: bolus dose n = 11 mice; constant dose n = 12 mice; escalating dose n = 11 mice. Data were combined from two independent experiments. Mean $\pm$ SEM is shown, and each data point represents an individual mouse. Kruskal-Wallis one-way ANOVA with Dunn’s multiple comparisons test was used for statistical evaluations. *p $\leq$ 0.05, **p$\leq$ 0.01.

**Reagents.**

| **Reagent** | **Source** | **Cat. #** |
| --- | --- | --- |
| Imject^TM^ Alum | Thermo Scientific | 77161 |
| Pneumococcal polysaccharide powder Type 14 (US Type 14) Pfizer (81-X) | ATCC | 81-X |
| Newborn calf serum | Gibco | 16-010-167 |
| Goat anti-mouse IgG-Fc HRP | Bethyl | A90-131P |
| Goat anti-mouse IgG1 HRP | Bethyl | A90-105P |
| Goat anti-mouse IgG2c HRP | Bethyl | A90-136P |
| KPL SureBlue TMB Microwell Peroxidase Substrate | SeraCare Life Sciences | 5120-0077 |
| Costar assay plate 96 well high binding | Fisher Scientific | 9018BC |
| Microplate 96 well PS half area high binding | Fisher Scientific | 675061 |
| Guanidine hydrochloride | Millipore Sigma | G3272 |
| IP Opaque Sterile hydrophobic high protein binding immobilon-P membrane plates | Millipore | MSIPS4W10 |
| Goat anti-mouse IgG-Fc Fragment | Bethyl | A90-131A |
| RPMI 1640 GlutaMAX Gibco | Gibco | 61870-036 |
| HI FBS | Gibco | 10082-147 |
| HEPES 1M | Gibco | 15630-080 |
| MEM Non-essential amino acids (100X) | Gibco | 11140-050 |
| Sodium pyruvate 100 mM | Gibco | 11360-070 |
| Pen Strep | Gibco | 15070-063 |
| B-mercaptoethanol 1000X | Gibco | 21985-023 |
| AEC substrate kit peroxidase | Vector Laboratories | SK-4200 |
| eBioscience Fixable Viability Dye eFluor 455UV | ThermoFisher Scientific | 65-0868-14 |
| Zombie Aqua Fixable Viability Kit | BioLegend | 77143 |
| Anti-mouse CD4-PerCP-Cy5.5 GK1.5 | BioLegend | 100434 |
| Anti-mouse B220-BV605 RA3-6B2 | BioLegend | 103244 |
| Anti-mouse CD279 (PD-1)-PE 29F.1A12 | BioLegend | 135206 |
| Anti-mouse CD185 (CXCR5)-biotin 2G8 | BD Pharmingen | 551960 |
| Anti-mouse CD95-PE-Cy7 Jo2 | BD Biosciences | 557653 |
| Anti-mouse GL7-Alexa488 GL7 | BioLegend | 144612 |
| Streptavidin-BV421 | BD Biosciences | 563259 |
| Anti-mouse Foxp3-Alexa Fluor 647 MF23 | BD Biosciences | 560401 |
| eBioscience Foxp3 / Transcription Factor Staining Buffer Set | ThermoFisher | 00-5523-00 |
| eBioscience, anti-mouse B220 rat IgG | ThermoFisher | 14-0460-82 |
| Rat anti-mouse/human GL-7 rat IgM | BioLegend | 144601 |
| CD4-biotin | BD Pharmingen | 553728 |
| Alexa Fluor® 647 AffiniPure™ Donkey Anti-Rat IgG (H+L) | Jackson ImmunoResearch | 712-605-153 |
| Alexa Fluor® 594 anti-rat IgM | BioLegend | 408912 |
| Streptavidin-AF488 | ThermoFisher | S11223 |
| Hoechst 33258 | ThermoFisher | H3569 |
